# Distribution of *Plasmodium* spp. infection in asymptomatic carriers in perennial and low seasonal malaria transmission settings in West Africa

**DOI:** 10.1186/s40249-018-0412-9

**Published:** 2018-04-25

**Authors:** Constant G. N. Gbalégba, Hampâté Ba, Kigbafori D. Silué, Ousmane Ba, Emmanuel Tia, Mouhamadou Chouaibou, Nathan T. Y. Tian-Bi, Grégoire Y. Yapi, Brama Koné, Jürg Utzinger, Benjamin G. Koudou

**Affiliations:** 1 Unité de Formation et de Recherche Sciences de la Nature, Université Nangui Abrogoua, 02 B.P. 801, Abidjan, 02 Côte d’Ivoire; 2 Centre Suisse de Recherches Scientifiques en Côte d’Ivoire , 01 B.P. 1303, Abidjan, 01 Côte d’Ivoire; 3 Laboratoire de Parasitologie – Mycologie, Institut National de Recherches en Santé Publique , B.P. 695, Nouakchott, Mauritania; 4 Unité de Formation et de Recherche Biosciences, Université Félix Houphouët-Boigny, 22 B.P. 582, Abidjan, 22 Côte d’Ivoire; 5 Centre d’Entomologie Médicale et Vétérinaire, Université Alassane Ouattara, 27 B.P. 529, Abidjan, 27 Côte d’Ivoire; 6 Université Péléforo Gon Coulibaly, B.P. 1328, Korhogo, Côte d’Ivoire; 7 Swiss Tropical and Public Health Institute , P.O. Box, CH- 4002 Basel, Switzerland; 8 University of Basel, P.O. Box, CH-4003 Basel, Switzerland; 9 Centre for Neglected Tropical Diseases, Liverpool School of Tropical Medicine , Pembroke Place, Liverpool, L3 5QA UK

**Keywords:** *Plasmodium* spp., Asymptomatic carriers, Urban area, Rapid diagnostic tests, Microscopy, Côte d’Ivoire, Mauritania, Korhogo, Kaedi

## Abstract

**Background:**

Since 2000, substantial progress has been made in reducing malaria worldwide. However, some countries in West Africa remain a hotspot for malaria with all age groups at risk. Asymptomatic carriers of *Plasmodium* spp. are important sources of infections for malaria vectors and thus contribute to the anchoring of the disease in favourable eco-epidemiological settings. The objective of this study was to assess the asymptomatic malaria case rates in Korhogo and Kaedi, two urban areas in northern Côte d’Ivoire and southern Mauritania, respectively.

**Methods:**

Cross-sectional surveys were carried out during the rainy season in 2014 and the dry season in 2015 in both settings. During each season, 728 households were randomly selected and a household-based questionnaire was implemented to collect demographic and epidemiological data, including of malaria preventive methods used in communities. Finger-prick blood samples were obtained for biological examination using microscopy and rapid diagnostic tests (RDTs).

**Results:**

Overall, 2672 households and 15 858 consenting participants were surveyed. *Plasmodium* spp. infection was confirmed in 12.4% (*n* = 832) and 0.3% (*n* = 22) of the assessed individuals in Korhogo and Kaedi, respectively. In Korhogo, the prevalence of asymptomatic malaria was 10.5% (95% *CI*: 9.7–11.2) as determined by microscopy and 9.3% (95% *CI*: 8.6–10.0%) when assessed by RDT. In Kaedi, asymptomatic malaria prevalence was 0.2% (95% *CI*: 0.1–0.4%) according to microscopy, while all RDTs performed were negative (*n* = 8372). In Korhogo, asymptomatic malaria infection was significantly associated with age and season, with higher risk within the 5–14 years-old, and during the rainy season. In Kaedi, the risk of asymptomatic malaria infection was associated with season only (higher during the dry season; crude *OR* (c*OR*): 6.37, 95% *CI*: 1.87–21.63). *P. falciparum* was the predominant species identified in both study sites representing 99.2% (*n* = 825) in Korhogo and 59.1% (*n* = 13) in Kaedi. Gametocytes were observed only in Korhogo and only during the rainy season at 1.3% (95% *CI*: 0.7–2.4%).

**Conclusions:**

Our findings show a low prevalence of clinical malaria episodes with a significant proportion of asymptomatic carriers in both urban areas. National policies for malaria infections are focused on treatment of symptomatic cases. Malaria control strategies should be designed for monitoring and managing malaria infections in asymptomatic carriers. Additional measures, including indoor residual spraying, effective use of long-lasting insecticidal nets is strongly needed to reduce the number of *Plasmodium* spp. infections in Korhogo and Kaedi.

**Electronic supplementary material:**

The online version of this article (10.1186/s40249-018-0412-9) contains supplementary material, which is available to authorized users.

## Multilingual abstracts

Please see Additional file 1 for translation of the abstract into the five official working languages of the United Nations.

## Background

Malaria remains an important challenge for public health and economic development across the African continent. More than 85% of clinical malaria episodes and 90% of malaria- related deaths occur in sub-Saharan Africa, mainly in young children [1]. A large proportion of *Plasmodium falciparum* infections are asymptomatic in endemic countries [2–5]. In some of these countries, *Plasmodium* spp. asymptomatic carriers are not yet targeted by national intervention strategies, and hence represent a persistent pool for maintaining the parasite life cycle and transmission by the anopheline vector [3]. As additional challenge, vectors seem to be more susceptible to parasites from asymptomatic carriers than from symptomatic one’s [6]. Consequently, the World Health Organization (WHO) suggests extending intervention strategies, including asymptomatic malaria cases, before planning for malaria elimination [1].

The commonly used criteria for defining asymptomatic malaria are parasite presence in peripheral thick blood films without symptoms and an axillary temperature less than 37.5 °C [2, 7–9]. However, parasite prevalence estimations vary considerably according to age group and performance of diagnostic test used [10]. Indeed, the quality of microscopic diagnosis is not satisfying in many sub-Saharan African settings [10–12].

In Côte d’Ivoire, malaria transmission occurs throughout the year with a peak during the rainy season. Malaria is the first cause (43%) of outpatient consultations in public health facilities [13]. The overall *Plasmodium* spp. prevalence rate through the country ranges from 64 to 75% [13]. In urban areas, malaria prevalence ranges from 30 to 40% [14]. However, the epidemiology of urban malaria in Côte d’Ivoire is not well documented, with a particular lack of information in the northern part of the country. Few studies pertaining to urban malaria were conducted in the South (Abidjan 2005, 2008), Southeast (Adzopé, in 2010) and the South-central (Taabo in 2016) of the country [15–18]. Data of the malaria burden in northern Côte d’Ivoire are more scarce. The most recent reports date back to 2003 and 2005 and identify the rural area of Korhogo district as hyper-endemic [19, 20]. The lack of recent published epidemiological data from Korhogo may be partly due to two Ivorian civil wars that interrupted education and research programme from 2002 to 2011.

In Mauritania, malaria is currently the main cause of morbidity (22%) and mortality (51%) in health facilities [21, 22]. The disease is the first cause of consultation and hospitalization in eight of thirteen regions of the country [22, 23]. An average of 200 000 malaria cases per year have been reported by the health centre information system [21, 24] and malaria represents the third most frequent reason for outpatient consultations after respiratory infections and diarrhoea [23, 25]. Similar to Côte d’Ivoire, Mauritania suffers from a lack of data at regional and national level. Epidemiological data on malaria are rare and out of date, except those for the capital Nouakchott [21, 23]. In addition, most of the malaria cases in prior studies were identified by presumptive diagnostic [24–26]. Based on climatic and geographic features, Mauritania is divided in three epidemiological strata; from the South to the North we have (i) the Sahelien zone, located along the Senegal River; (ii) the Sahelo-saharan zone; and (iii) the Sahara zone, the largest one. In Kaedi, city that is located in the Sahelien zone and where *P. falciparum* is endemic, 25% of morbidity and 39% of mortality are caused by malaria according to the Ministry of Health [25]. A recent study conducted across these strata revealed that the Sahelo-sahara zone had the highest malaria burden, while the Sahelien zone had the lowest malaria prevalence [24]. However, the parasitological investigations were limited to 2–9 years-old children. Clinical data on asymptomatic *Plasmodium* carriers in Mauritania are even harder to find. In the Gorgol region, where Kaedi is the main city, malaria transmission is not well documented [23, 27]. Health authorities entertained hopes of reducing malaria morbidity and mortality by 75% by 2015 and potentially eliminate the disease from Mauritania [25]. In this context of low and seasonal malaria transmission, prevalence of asymptomatic infections needs to be clearly documented and appropriate interventions implemented for their adequate management.

Presently, in both countries, national malaria control programme strategies are based on (i) appropriate diagnosis and effective treatment of clinical cases; (ii) distribution of insecticide-treated nets (ITNs) free of charge among population, and (iii) intermittent preventive treatment (IPT) during pregnancy [13, 24]. However, larval control and indoor residual spraying (IRS) have not yet been implemented.

To enhance understanding of the epidemiology of asymptomatic malaria infection in these urban areas, two cross-sectional surveys were carried out for each site in two consecutive years (2014 and 2015) during the rainy and dry seasons.

## Methods

### Study sites

**Fig. 1:**
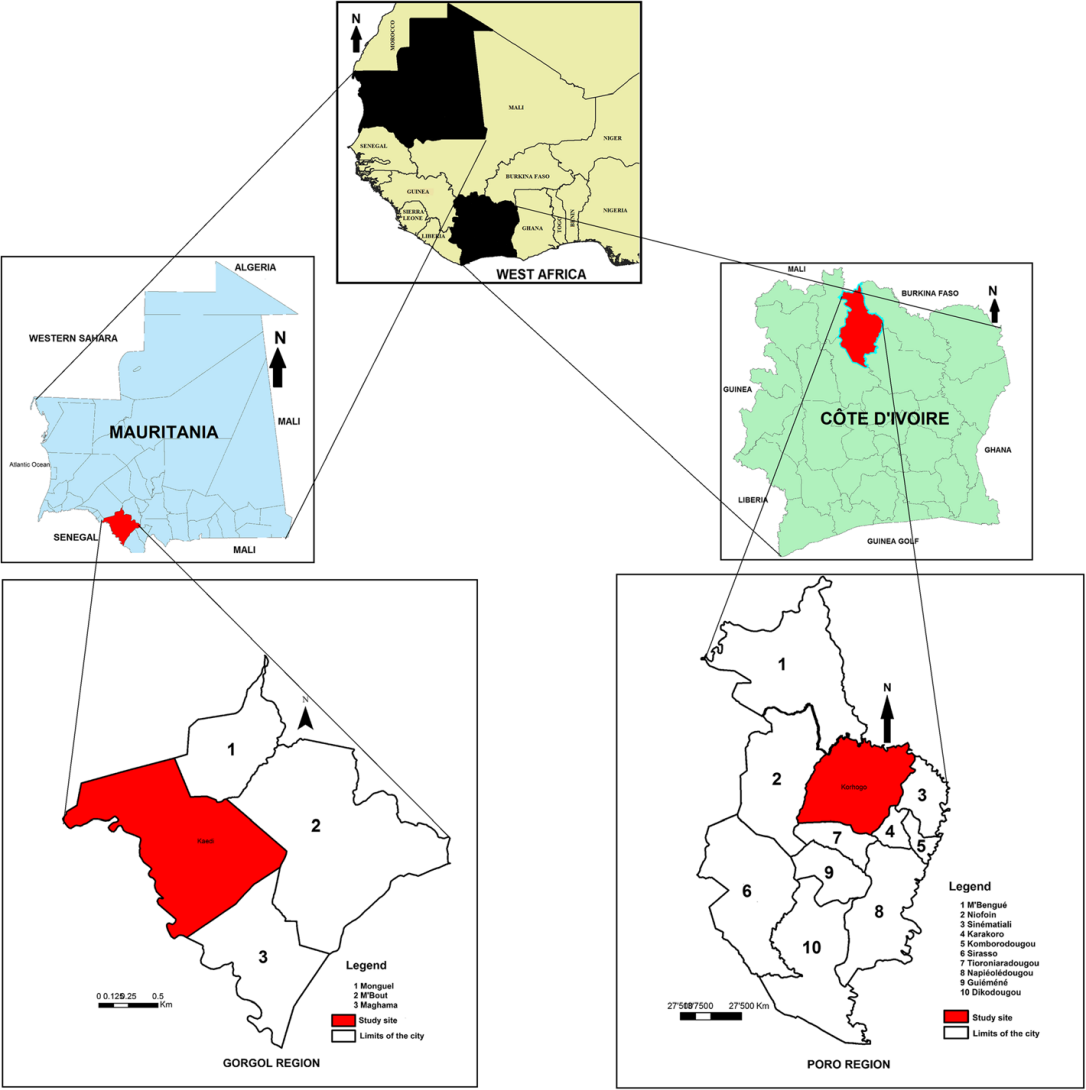
Localization of study sites in Côte d’Ivoire and the Islamic Republic of Mauritania

This study was conducted in urban areas of Korhogo in northern Côte d’Ivoire (N 09°27′41″; W 05°38′19″) and Kaedi in southern Mauritania (N 16°09′02″; W 13°30′20″) (Fig. 1). Korhogo is located at 663 km from Abidjan, the economic capital of Côte d’Ivoire. Its population was estimated at 286 076 inhabitants in 2014 [28]. The average annual temperature is 27 °C and the average annual precipitation ranges from 1000 to 1300 mm [29]. The climate is Sudanese [30], characterized by one dry season (November–April) and one rainy season (May–October) with two rainfall peaks in June and September. The city has a water supply dam (flow 10^6^ m^3^) around which urban gardening has developed. Korhogo has a regional hospital with a bed capacity of 372 patients, a tuberculosis and blood transfusion centre.

Kaedi was already described elsewhere [31]. Briefly, the city is located approximately at 435 km from Nouakchott, the capital of Mauritania, and lies along the Senegal River that marks the border between Mauritania and Senegal. In 2013, its population was estimated at 118 195 inhabitants. The average annual temperature is 29.5 °C and the average annual rainfall ranges from 300 to 500 mm. Kaedi is located within a Sahelian type climate zone with a wet season from July to October, a ‘cool’ dry season from November to March and a ‘hot’ dry season from March to June. Kaedi has a regional hospital with a bed capacity of 120 patients.

The choice of the two urban settings is justified by the following reasons: (i) their respective positions to the North and South of the strip of the Sahel; (ii) because they were the study sites of a former project (Canadian International Development Research Centre [IDRC], grant no. 104270–013), which permit the establishment of research teams in both countries; (iii) the two study sites are secondary cities, even more vulnerable to the negative effects of climate change because of basic infrastructure (compared to capital cities) in most developing countries; (iv) and over the past 30–40 years, West Africa region has been characterized by a decline of rainfall and the increasing of 1 °C in air temperature. In this context, high climatic variability conditions were observed in the two cities (i.e. intense periods of droughts [2007] and floods [2010, 2012]). Of note malaria is endemic and constitute public health problem in both cities.

### Study design, population and procedures

Cross-sectional surveys were carried in July 2014 and March 2015 (in Korhogo) and in September 2014 and May 2015 (in Kaedi), during the rainy and the dry season, respectively.

For each survey, households were randomly selected from the 29 and 11 demographic zones in Korhogo and Kaedi, respectively. The number of households by demographic zones was proportionally allocated according to population size in each zone. The sample size (*n*) was adjusted to 728 households obtained by using the following formula:

$n=\frac{\delta^2\times P\left(1-P\right)\times C}{i^2}$ where *δ*, standard deviation (1.96); *P*, the expected prevalence (35%) based on [32]; *i*, precision or margin of error (5%) and *C* = 2, correction coefficient.

In each site, the objective of the study was discussed with the selected healds of household. For each household head or their representative, written informed consent was obtained and a questionnaire was administered in order to obtain demographic information of all household members as well as malaria preventive measures used in the household. Thick and thin blood films and rapid diagnostic tests (RDTs) were systematically performed for all consenting and assenting participants across all age groups. In addition, weight and axillary temperature were recorded. In each household, clinical malaria cases based on RDT positive + fever (because microscopy readings were not yet available) were treated with artemisinin-based combination therapy (ACT), according to country guidelines. In Côte d’Ivoire, clinical cases received artesunate and amodiaquine according to age. Treatments were performed by physician/nurses from the regional hospital of Korhogo. In Mauritania, clinical cases were not treated as all RDT were negative.

### Laboratory evaluation

Based on the predominant *Plasmodium* species encountered at each study site, malaria Ag P.f (Standard Diagnostics (SD) Inc.; Yongin, Republic of Korea) was used in Korhogo. Diagnostic tests based on histidine-rich protein 2 are specific to *P. falciparum.* The Pf/HRP2-based RTDs are recommended by the national malaria control program and used at national level in Côte d’Ivoire. In Kaedi, Ag P.f/Pan (SD, Inc.; Yongin, Republic of Korea) was applied for detection of *Plasmodium* spp. These tests are recommended by the national malaria control programme in Mauritania for simultaneous detection and differentiation of HRP2 and pLDH (*Plasmodium* lactate dehydrogenase) specific to other *Plasmodium* species in human specimens. RDT results were obtained within 15 min during the household visit. Finger prick blood samples were taken from study participants and labelled with the same participant code. Thin blood films were fixed with methanol. Thick and thin blood films were stained with 10% Giemsa and examined under a microscope at × 100 magnitude with immersion oil. Microscope readings were performed at the “Centre Suisse de Recherches Scientifiques en Côte d’Ivoire” and the “Institut National de Recherche en Santé Publique, Nouakchott” by experienced technicians under the supervision of a parasitologist. Thin blood films were examined for *Plasmodium* species identification and asexual stage counted according to 200 or 500 leucocytes. Parasite density was expressed as the number of asexual parasites per μl of blood [33]. A quality control was made for 10% randomly selected slides by a senior technician. Slides with conflicting results were re-read by a third technician and the results discussed until a consensus was reached.

### Data analysis

Data were entered with Epidata (EpiData Association; Odense, Denmark) and analyzed using Stata version 14.1 (Stata Corporation; College Station, TX, USA). Prevalence of infection was estimated as proportion and compared using Pearson χ2 test or Fisher’s exact test, as appropriate. Data summary was done using descriptive statistics. Parasite density was normalized by log (x + 1) for a 0 count and to obtain similar variances between groups before using parametric tests. ANOVA one way or *t*–test was used to check for any statistical difference in the geometric means of parasite density between sex, age-groups and season and a 95% *CI* was used to measure the strength of association between prevalence or parasite density infection status and variables. Fever was defined as having an axillary body temperature that is equal to or greater than 37.5 °C and clinical malaria was defined as association of fever and the presence of asexual parasites of *Plasmodium* spp. in the blood film [34]. Age was stratified into six groups: < 1; 1–4; 5–9; 10–14; 15–19 and ≥ 20 years, according WHO guidelines [35]. *Plasmodium* spp. infection intensity was categorised by three groups: 1–500, 501–5000 and > 5000 parasites/μl of blood. Bivariate analyses were used to evaluate association between estimates prevalence and sex, age and season. Binary logistic regression was employed to assess the risk factors associated with *Plasmodium* spp. infection. Variables significant at *P*-value of 0.25 in the univariate logistic regression were selected for multivariate logistic regression analysis model. For all statistical tests, a 5% level of significance level was used.

## Results

### Characteristics of the study participants

**Fig. 2:**
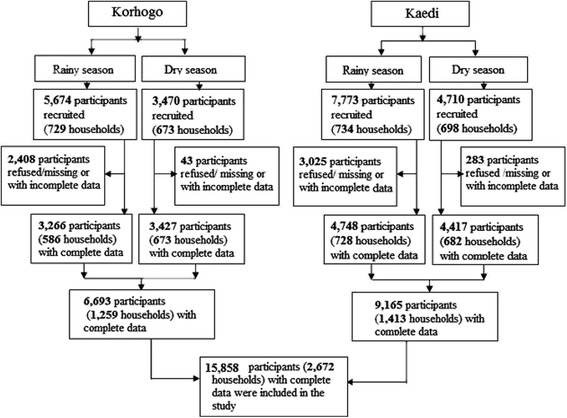
Study flow diagram showing participant enrolment and diagnostics performed in Korhogo, northern Côte d’Ivoire and Kaedi, southern Mauritania during the rainy and dry seasons, 2014/2015

Information on the season and demographic characteristics of the study participants are summarized in Table 1. In Korhogo, a total of 1259 households were included in the study (Fig. 2) with a total of 9144 individuals of which 6693 (73.2%) had a complete data set. Most of study participants (*n* = 3006/6693; 76.1%) were illiterate (*n* = 1518; 38.4%) or had attended primary school (*n* = 1488; 37.7%). In Kaedi, a total of 1413 households were included in the study (Fig. 2) with a total of 12 488 individuals of which 9165 (73.2%) had complete data. The majority of the individuals included in the study were illiterate (*n* = 3561, 40.3%), or had primary school level (*n* = 2901; 32.8%).

**Table 1:** Demographic characteristics of the sample populations in Korhogo, northern Côte d’Ivoire and Kaedi southern Mauritania, 2014/2015

Variable	Korhogo		Kaedi	
	Frequency (%)	*P*–value	Frequency (%)	*P*–value
Participants with complete data	6693 (73.2)		9165 (73.4)	
Sex				
Male	2801 (41.8)	< 0.001	3612 (39.4)	< 0.001
Female	3892 (58.2)		5553 (60.6)	
Sex ratio (male:female)	0.72		0.65	
Age group (year)				
< 1	223 (3.3)	< 0.001	436 (4.8)	< 0.001
1–4	925 (13.8)		1115 (12.2)	
5–9	1201 (18.0)		1908 (20.8)	
10–14	962 (14.4)		2201 (24.0)	
15–19	710 (10.6)		1997 (21.8)	
≥ 20	2672 (39.9)		1508 (16.5)	
Education level				
Illiterate	1518 (38.5)	< 0.001	3561 (40.3)	< 0.001
Koranic school	238 (6.0)		1188 (13.4)	
Primary school	1488 (37.7)		2901 (32.8)	
Secondary school	628 (15.9)		1070 (12.1)	
Higher academic level	75 (1.9)		126 (1.4)	
Possession of LLINs				
Yes	1308 (93.2)	< 0.001	1391 (95.7)	< 0.001
No	95 (6.8)		62 (4.3)	
Season				
Rainy	3266 (48.8)	< 0.001	4748 (51.8)	< 0.001
Dry	3427 (51.2)		4417 (48.2)	

*LLIN* Long-lasting insecticidal nets, *P*-value calculated Pearson’s χ2 test for groups within the different settings

The rate of long-lasting insecticidal nets (LLINs) possession in selected households of the two study sites was 93.2% (1308/1403) in Korhogo and 95.7% (1391/1453) in Kaedi.

### *Plasmodium* spp. infection prevalence and parasite density

The prevalence of *Plasmodium* spp. infection and parasite density according to season and demographic characteristics are presented in Table 2.

**Table 2:** *Plasmodium* spp. prevalence by microscopy and parasite density among study participants in Korhogo, northern Côte d’Ivoire and Kaedi southern Mauritania, 2014/2015, stratified by sex, age, fever status and season

Variable	Korhogo			Kaedi		
	*n* (Positive)	Prevalence (95% *CI*)	GMPD (95% *CI*)	*n* (Positive)	Prevalence (95% *CI*)	GMPD (95% *CI*)
Overall	6693 (832)	12.4 (11.7–13.2)	220 (199–244)	9165 (22)	0.2 (0.1–0.4)	229 (153–357)
Sex						
Male	2801 (370)	13.2 (12.0–14.5)	256 (223–295)^b^*	3612 (8)	0.2 (0.1–0.4)	199 (93–482)^b^
Female	3892 (462)	11.9 (10.9–12.9)	195 (171–223)	5553 (14)	0.4 (0.1–0.4)	249 (144–449)
Age group (year)						
< 1	223 (22)	9.9 (6.6–14.6)*	158 (96–275)^c^*	175 (0)		
1–4	925 (111)	12.0 (10.1–14.3)	275 (205–375)	1376 (7)	0.5 (0.2–1.0)	275 (99–897)^c^
5–9	1201 (197)	16.4 (14.4–18.6)	261 (212–326)	1908 (3)	0.2 (0.0–0.4)	180 (56–787)
10–14	962 (160)	16.6 (14.4–19.1)	223 (178–280)	1301 (2)	0.2 (0.0–0.6)	651 (400–857)
15–19	710 (89)	12.5 (10.3–15.2)	227 (170–307)	900 (1)	0.1 (0.0–0.6)	436 (29-465)
≥ 20	2672 (253)	9.5 (8.4–10.6)	178 (152–210)	3505 (9)	0.3 (0.1–0.5)	165 (97–292)
Fever^a^						
Yes	538 (83)	15.4 (12.6–18.7)^d^*	329 (229–487)^b^*	522 (1)	0.2 (0.0–1.1)	3497 (2072-4284)^b^
No	5890 (673)	11.4 (10.6–12.3)	208 (188–232)	8372 (21)	0.3 (0.2–0.4)	205 (143–304)
Season						
Rainy	3266 (567)	17.4 (16.1–18.7)^d^*	236 (210–264)^b^*	4748 (3)	0.1 (0.0–0.2)^d^*	320 (32–608)^b^*
Dry	3427 (265)	7.7 (6.9–8.7)	191 (160–229)	4417 (19)	0.4 (0.3–0.7)	218 (146–336)

^a^Out of study participants, 265 (4%) and 271 (3%) did not have axillary temperature data in Korhogo and Kaedi, respectively; ^b^Obtained by Student’s *t*-test between groups, ^c^Obtained by ANOVA between groups; ^d^Obtained by Pearson’s χ2 test; * *P* < 0.05; *CI* Confidence interval; *GMPD* Geometric mean of parasite density (parasites/μl); *n* Number examined

In Korhogo, 6693 microscopy diagnoses on thick blood films and an equivalent number of RDTs was carried out during the two seasons in parallel. The overall *Plasmodium* parasite rate in both seasons as confirmed by microscopy was 12.4% (95% *CI*: 11.7–13.2%), while the rate covered with the RDT was 10.5% (95% *CI*: 9.8–11.3%). The parasite index was 17.4% (95% *CI*: 16.1–18.7%) for the rainy season and 7.7% (95% *CI*: 6.9–8.7%) for the dry season. *P. falciparum* prevalence (RDT positive) was 11.7% (95% *CI*: 10.7–12.9%) and 9.3% (95% *CI*: 8.4–10.4%), during the rainy and dry season, respectively. *Plasmodium* spp. infection was significantly associated with age group, fever status and the season (Table 2). Moreover, the prevalence of infection peaked in age group 10–14 years using each of the diagnostic methods. Febrile participants had significantly higher (15.4%; 95% *CI*: 12.6–18.7%) *Plasmodium* spp. prevalence compared to non-febrile participants (11.4%; 95% *CI*: 10.6–12.3%) (*P* = 0.006). Similarly, the prevalence of *Plasmodium* spp. was significantly higher during the rainy season compared to the dry season for both diagnostic methods (*P* <  0.001 for both). The overall geometric mean of parasite density (GMPD) among infected participants was 220 parasites/μl (95% *CI*: 199–244). Parasite density was sex, age, fever status and season dependant (Table 2). Indeed, the GMPD of male (256 parasites/μl, 95% *CI*: 223–295) was higher compared to female (195 parasites/μl, 95% *CI*: 171–223). The GMPD was relatively high in 1–4 years-old children (275 parasites/μl, 95% *CI*: 205–375) and low in adult participants (178 parasites/μl, 95% *CI*: 152–210), similarly among the febrile (329 parasites/μl, 95% *CI*: 229–487) compared with non-febrile participants (208 parasites/μl, 95% *CI*: 188–232). The rainy season had the highest GMPD (236 parasites/μl, 95% *CI*: 210–264). *P. falciparum*, *P. malariae* and mixed infections accounted for 99.2% (825/832), 0.2% (2/832) and 0.6% (5/832) of the total cases, respectively. No *P. ovale* infection was observed in any of the examined blood samples. Eleven individuals (11/825; 1.3%) carried gametocytes during the rainy season (Table 3). The prevalence rate of *P. falciparum* gametocytes was found to be similar in terms of sex, age and fever status. No gametocytes were found in individuals aged less than 1 year and between 15 and 19 years-old.

**Table 3:** *Plasmodium falciparum* gametocytes among the study population, stratified by sex, age, parasite density and season in Korhogo, northern Côte d’Ivoire, 2014/2015

Variable	*P. falciparum* gametocytes				
	*n*	Positive	Prevalence (95% *CI*)	χ^2^	*P-*value
Overall	825	11	1.3 (0.7–2.4)		
Sex					
Male	365	5	1.4 (0.5–3.2)	11.3	0.255
Female	460	6	1.3 (0.5–2.8)		
Age group (year)					
< 1	21	0	–	47.23	0.381
1–4	110	2	1.8 (0.2–6.4)		
5–9	197	3	1.5 (0.3–4.4)		
10–14	156	3	1.9 (0.4–5.5)		
15–19	89	0	–		
≥ 20	252	3	1.2 (0.3–3.4)		
Fever^a^					
Yes	82	1	1.2 (0.0–6.6)	11.95	0.153
No	668	10	1.5 (0.7–2.7)		
PD (parasites/μl of blood)					
1–500	577	12	2.1 (1.1–3.6)	3.19	0.202
501–5000	207	1	0.5 (0.0–2.7)		
> 5000	41	0	–		
Season					
Rainy	560	11	1.9 (1.0–3.5)	13.66	0.135
Dry	265	0	–		

^a^Of the participants examined for *P. falciparum* gametocytes, 75 (10.6%) did not have axillary temperature data. *CI* Confidence interval, *PD* Parasite density, *n* Number examined

In Kaedi, 9165 thick and thin blood films and an equivalent number of RDTs (SD Bioline–Antigen Pf/Pan) were performed during both seasons. All RDTs were negative. The prevalence of *Plasmodium* spp. infection, as assessed by microscopy across both seasons, was 0.2% (95% *CI*: 0.1–0.4%) (Table 2). This prevalence was similar in all age groups. In addition, no infants under 1 year of age were infected. *Plasmodium* spp. infection was similar in febrile and non-febrile participants. However, the prevalence of infection was higher in the dry season (0.4%, 95% *CI*: 0.3–0.7%) compared to the wet season (0.1%, 95% *CI*: 0.0–0.2%), (*χ*^*2*^ = 12.90, *P* <  0.001). The overall GMPD among infected participants was 229 parasites/μl (95% *CI*: 153–357). The GMPD was relatively high in 10–14 years-old children (651 parasites/μl, 95% *CI*: 400–857) and low in adult participants (165 parasites/μl, 95% *CI*: 97–292). Season was significantly associated with parasite density, as assessed by bivariate analysis (Table 2). The GMPD was higher in the dry season than the rainy season (*OR*: 6.82, 95% *CI*: 2.02–23.10) with a more than six times higher odds to carry a parasite in this season. Sex, age and fever status were not significantly associated with parasite density. Three species of *Plasmodium* were reported: *P. falciparum* (*n* = 13, 56.5%), *P. malariae* (*n* = 2, 8.7%) and *P. vivax* (*n* = 1, 4.3%). In subsequent  analyses, the only case of *P. vivax* was not included due to absence of sex and age information. In addition, in 7 infections (30.4%), *Plasmodium* spp. could not be identified due to the deterioration of the thin blood film.

### Prevalence, parasite density and factors associated with asymptomatic *Plasmodium* spp. infection

The prevalence, parasite density and the factors associated with asymptomatic *Plasmodium* spp. infection according to setting, season and demographic characteristics are presented in Table 4.

**Table 4:** Prevalence by microscopy, parasite density and factors associated with asymptomatic *Plasmodium* spp. infection among study participants in Korhogo, northern Côte d’Ivoire and in Kaedi, southern Mauritania, 2014/2015

Variable	Korhogo					Kaedi			
	*n* (Positive)	Prevalence (95% *CI*)	Univariate c*OR* (95% *CI*)	Multivariate a*OR* (95% *CI*)	GMPD (95% *CI*)	*n* (Positive)	Prevalence (95% *CI*)	Univariate c*OR* (95% *CI*)	GMPD (95% *CI*)
Overall	6428 (673)	10.5 (9.7–11.2)			208 (188–232)	8894 (21)	0.2 (0.1–0.3)		208 (144–307)
Sex									
Male	3744 (302)	8.1 (7.2–9.0)	1.00	1.00	249 (214–292)^a^*	3494 (7)	0.2 (0.1–0.4)	1.00	146 (109–197)^a^
Female	2684 (371)	13.8 (12.5–15.2)	1.16 (0.98–1.36)^d^	1.06 (0.90–1.26)	182 (158–210)	5400 (14)	0.3 (0.1–0.4)	1.29 (0.52–3.20)	249 (146–449)
Age group (year)									
< 1	211 (16)	7.6 (4.4–12.0)^c^*	1.00	1.00	113 (69–193)^b^*	167 (0)	–	–	–
1–4	887 (84)	9.5 (7.6–11.6)	0.77 (0.44–1.35)	0.79 (0.45–1.40)	254 (182–360)	1323 (6)	0.5 (0.2–1.0)	170 (0.60–4.80)	197 (91–482)^b^
5–9	1157 (163)	14.1 (12.1–16.2)	0.49 (0.29–0.84)^d^	0.51 (0.30–0.87)*	264 (210–339)	1853 (3)	0.2 (0.0–0.5)	0.61 (0.16–2.26)	180 (57–787)
10–14	917 (127)	13.8 (13.1–16.3)	0.49 (0.28–0.85)^d^	0.50 (0.30–0.87)*	190 (152–236)	1265 (2)	0.2 (0.0–0.6)	0.60 (0.13–2.79)	651 (400–857)
15–19	684 (73)	10.7 (8.5–13.2)	0.69 (0.39–1.22)^d^	0.71 (0.40–1.27)	229 (166–323)	873 (1)	0.1 (0.0–0.6)	0.43 (0.05–3.43	436 (29-465)
≥ 20	2572 (210)	8.2 (7.1–9.3)	0.94 (0.55–1.60)	0.99 (0.58–1.70)	175 (147–208)	3413 (9)	0.3 (0.1–0.5)	1.00	166 (98–295)
Season									
Rainy	3167 (446)	14.1 (12.9–15.3)^c^*	0.45 (0.38–0.54)^d^	0.45 (0.38–0.53)*	212 (188–241)^a^*	4633 (3)	0.1 (0.0–0.2)^c^*	1.00	323 (144–455)^a^*
Dry	3261 (227)	7.0 (6.1–7.9)	1.00	1.00	201 (166–246)	4261 (18)	0.4 (0.3–0.7)	6.36 (1.87–21.63)^d^	193 (140–272)

^a^Obtained by Student’s *t*-test between groups; ^b^obtained by ANOVA between groups; ^c^obtained by Pearson’s χ2 test between groups, ^d^Significant at *P*-value < 0.25; a*OR* Adjusted odds ratio, c*OR* Crude odds ratio, *CI* Confidence interval, *GMPD* Geometric mean of parasitaemia, *n* Number examined; *significant at *P*-value < 0.05

**Fig. 3:**
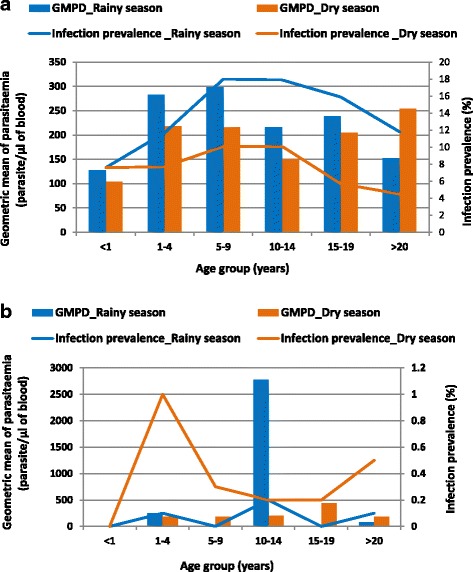
Age-specific prevalence of asymptomatic *Plasmodium* spp. infection and geometric mean parasitaemia within the study participant stratified by season (**a**) in Korhogo (northern Côte d’Ivoire) and (**b**) in Kaedi (southern Mauritania), 2014/2015

In Korhogo, the overall prevalence of asymptomatic malaria was 10.5% (95% *CI*: 9.7–11.2%). Females were more affected with asymptomatic infection than males but the difference was not statistically significant (*χ*^*2*^ = 3.15, *P* = 0.076). Asymptomatic infection was observed in all age groups. Children aged 5–9 years (14.1%, 95% *CI*: 12.1–16.2%) and 10–14 years (13.8%, 95% *CI*: 13.1–16.3%) were the most likely to be infected, while infants less than 1 year were found to be the least infected (7.6%, 95% *CI*: 4.4–12.0%) (Fig. 3). Moreover, it was found that the prevalence of asymptomatic infection was higher during the rainy season compared to the dry season (*χ*^*2*^ = 86.97, *P* = 0.001).

The GMPD was 208 parasites/μl of blood (95% *CI*: 188–232). Parasite density was associated with sex, age and season. Indeed, it was higher in males (GMPD: 249 parasites/μl, 95% *CI*: 214–292) than in females (GMPD: 182 parasites/μl, 95% CI: 158–210), *(t* = 2.21, *P* = 0.013). Children aged 5–9 years presented the highest GMPD (264 parasites/μl, 95% *CI*: 210–339) (Fig. 3). The GMPD was also higher in the rainy season (212 parasites/μl, 95% *CI*: 188–241) compared to the dry season (201 parasites/μl, 95% *CI*: 166–246) (*t* = − 11.99, *P* <  0.001).

In Kaedi the overall prevalence of asymptomatic infection was 0.2% (95% *CI*: 0.1–0.3%) with males and females having the same probability of being infected with asymptomatic infection (*χ*^*2*^ = 0.30, *P* = 0.580). All age groups were affected by asymptomatic malaria, except infants under 1 year old. The prevalence of asymptomatic infection was statistically similar in all age groups, with children aged 1–4 years being the most infected (Fig. 3).

Asymptomatic infections were significantly associated with season and was more likely to occur in the dry season compared to the wet season (*χ*^*2*^ = 11.60, *P* = 0.001).

The overall GMPD was 208 parasites/μl of blood (95% *CI*: 144–307). Females had a higher GMPD (249 parasites/μl, 95% *CI*: 146–449) compared to males (146 parasites/μl, 95% *CI*: 109–197); however, the difference was not statistically significant. Children aged 10–14 years had the highest GMPD (651 parasites/μl, 95% *CI*: 400–857) (Fig. 3). The GMPD was higher in the rainy season (323 parasites/μl, 95% *CI*: 144–455) compared to the dry season (193 parasites/μl, 95% *CI*: 140–272), (*t* = − 3.45; *P* = 0.003).

In the univariate analysis, sex, age group and season were significantly associated with the prevalence of asymptomatic *Plasmodium* spp. infection in Korhogo at *P*-value < 0.25. However, in the multivariable logistic regression analysis, age group 5–9 years (adjusted *OR)*, [a*OR*]: 0.51, 95% *CI*: 0.30–0.87), 10–14 years (a*OR:* 0.50, 95% *CI*: 0.30–0.87) and the rainy season (a*OR:* 0.45, 95% *CI*: 0.38–0.53) were significantly associated with the prevalence of asymptomatic *Plasmodium* spp. infection.

In Kaedi, by contrast, only the season was associated with asymptomatic *Plasmodium* spp. infection, this reason did not allow us to perform a multivariate analysis. In univariate analysis, the risk of asymptomatic *Plasmodium* spp. infection was 6.36 times (crude *OR* [c*OR*]: 1.87–21.63) higher during the dry season, compared to the rainy season.

## Discussion

The present study assessed the prevalence of *Plasmodium* spp. with a focus on the distribution of *Plasmodium* spp. infection in asymptomatic carriers from urban areas of Korhogo in Côte d’Ivoire and Kaedi in Mauritania.

The prevalence of *Plasmodium* infection observed in Korhogo (12.4%) was generally low compared to other reports in urban settings in Côte d’Ivoire [15, 18, 36]. A particularly low prevalence was observed in participants under 1 year and between 1 and 4 years of age, which is likely to be due to the protective effect of maternal antibodies against malaria antigens [37–39]. As the protective >effect of maternal antibodies decreases with age, the observed low prevalence over this age group may also be related to the integrated management of less than 5 years-old patients. Efforts have been made to improved health system in the management of diseases in this age group.

The low prevalence of malaria infection observed in Kaedi (0.2%, 22/9165) may be partly attributed to the divers Global Fund-supported intervention strategies implemented in this area, such as LLINs, IPT for pregnant women and immediate treatment with ACTs. In a recent study conducted in Rosso, a city located along the Senegal River, localised in the Sahelian zone, Ouldabdallahi and colleagues [40] reported a higher prevalence rate. In this city, 2.5% of the 1431 febrile paediatric and adult patients screened were malaria-positive. Indeed, the zone bordering the Senegal River has a general reputation to be a high *P. falciparum* setting [24]. Nevertheless, malaria prevalence was low in the Sahelian zone, whilst the highest burden was observed in the Sahelo-Saharan zone.

A recent study conducted in three strata in Mauritania [24] showed that the Sahelian zone had the lowest malaria prevalence (14/1056; 1.3%), compared to the other strata. The situation was shown to be similar at the opposite site of the River, where the number of new malaria cases was low since 1990 [41, 42].

School-aged children (i.e. 5–14 years of age) in Korhogo and preschool-aged children (< 5 years) in Kaedi presented the highest asymptomatic malaria prevalence. It was previously shown that teenagers sleep under bed nets less frequently than the preschool-aged children. Those asymptomatic teenagers raise many issues because they are unknown carriers of the parasite and these reservoirs contribute in maintaining the parasite lifecycle, as documented in malaria-endemic communities [37, 43]. However, African teenagers often show clinical immunity that prevents them to express malaria symptoms in spite of their regular exposure to the parasite during seasons of high-transmission [37]. Prevention and treatment programme rarely target school aged-children as opposed to younger children that leave an important intervention gap. School-aged children need to be included in any comprehensive malaria prevention strategy.

In the current study, parasitaemia increased with age in children aged 1–5 years, and declined thereafter, suggesting a certain immune protection during the childhood obtained at an early age. This explains why children falling into this age category were found to be asymptomatic most of the times.

The proportion of *Plasmodium* spp. positive febrile participants was 15.4% and 0, 2% in Korhogo and Kaedi, respectively. The prevalence of *Plasmodium* spp. in feverish participants in Korhogo was higher than in those with no fever. Indeed, the majority of fever cases in both sites was not due to malaria parasitaemia. The association between malaria parasitaemia and fever has been convincingly documented in endemic areas [44–46]. In urban areas, fevers are not necessarily due to malaria; hence, treatment of fever as malaria based only on a presumptive diagnostic could be considered as wastage of resources and lead to sub-optimal or wrong treatment. Unfortunately, malaria presumptive treatment is common in health facilities in the study sites and elsewhere in the two countries [13, 22].

The prevalence of asymptomatic infection in Korhogo was 10.5% (ranging from 8.3% to 12.3%). This prevalence rate was similar to the prevalence of asymptomatic malaria cases documented in a mountainous district in Ghana (11.9%) [47]. This finding showns a lower percentage than the one reported from urban setting elsewhere in Côte d’Ivoire [18, 36] but was higher than the prevalence reported in urban areas of Dakar (ranging from 0% to 7.5%) [48] and Kenya (ranging from 1.3% to 8.1%) [49]. In Kaedi, the prevalence of asymptomatic infection was 0.2% (ranging from 0.1% to 0.3%) and the majority of participants with a positive blood film (21/22; 95%) were asymptomatic. Therefore, this prevalence is low compared with that obtained in a study conducted among school-aged children (*n* = 1040, 0.9%) in Trarza region in the Sahelian zone [40]. Asymptomatic infection cases were likely to be detected more by microscopy, mainly in Kaedi, compared to RDTs. RDTs are often less sensitive in low parasitaemia (< 100 parasites/μl of blood). In addition, mutation can occur in parasite strain. *Pf-HRP2* mutations reported in Central and South America and Asia [50, 51] have yet to be documented in our study areas. These mutations can have an impact on RDT sensitivity.

In areas where malaria transmission is high, regular infection with *Plasmodium* results in partial immunity and favours asymptomatic parasite carriers (APCs) [2, 48, 52]. These APCs represent parasites reservoir and contribute to disease distribution by infection of anopheline mosquitoes in some period [53–55]. These periods could be the rainy season in Korhogo, and specifically, two months after the irrigation of the rice fields in Kaedi. Therefore, the treatment of APCs in endemic areas may reduce the rate of clinical cases [56].

In the two sites surveyed, malaria infection follows a seasonal variation. However, while in Korhogo malaria was found at the highest prevalence during the rainy season, the opposite was observed in Kaedi where the prevalence of infection was higher during the dry season. In Korhogo, the high malaria prevalence during the rainy season is likely to be due to the abundance of *Anopheles* mosquitoes during this period. The rainfall creates more productive breeding sites to renew vectors populations, and hence, malaria transmission intensifies. Moreover, during the rainy period, higher amounts of gametocyte carriers are generated. The observations made in Kaedi do not fit with this common pattern and are different to a report from a southern area of Mauritania [24], where malaria prevalence was found to be higher during the rainy season, compared with the 'cool' dry season. This contradictory result could be due to the period of field investigations and the low abundance of malaria vectors during this period. A previous study [57] recorded a prevalence rate of 16.6% in April–May 2003 in Nouakchott, corresponding to the dry season, however, the study participant were febrile when they were recruited. A recent study conducted in Kaedi, during the dry season, revealed the absence of *Plasmodium* parasitaemia [26]. However, only children aged between 6 and 59 months were screened. Socio-economic environment and geographic characteristics of a town seem to influence both malaria transmission and severity. Also, vectors densities and sporozoite rates influence entomological inoculation rate.

In Korhogo, two *Plasmodium* species were found (i.e. *P. falciparum* and *P. malariae*) out of the three species previously reported from Côte d’Ivoire [18, 36]. In Kaedi, three species of *Plasmodium* were observed during our study (i.e. *P. falciparum*, *P. malariae* and *P. vivax*) as previously reported from Mauritania [21, 24, 58] and the Sahelian zone along the Senegal River in particular. However, only one case of *P. vivax* was found. This species is mainly found in Nouakchott because of a high density of Duffy-positive *P. vivax*-susceptible individuals among the population [21, 23]. A recent study conducted in the Sahelian zone did not find any *P. vivax* infection among 1050 children screened [24]. Our single *P. vivax* identified case may be related to people movement from Nouakchott, *P. vivax* endemic area in Mauritania. *P. falciparum* was the dominant species in Korhogo and in Kaedi, which is in line with previous studies in both countries [18, 21, 26, 36] and in West Africa [47, 59]. Yet, WHO indicates in the most recent malaria country profile map for Mauritania, that the Gorgol region is free of *P. falciparum* cases [60]. Data generated from this study can greatly contribute to update regional epidemiological data over years.

Our study has several limitations that should be taken into consideration when interpreting the results. Firstly, the current level of APCs proportion, especially in Kaedi, may be underestimated. Indeed, in low transmission area, parasite density seemed low; therefore, high sensibility diagnostic methods like PCR and loop-mediated isothermal DNA amplification are needed to identify malaria cases that are not detectable via microscopy [55, 61]. Furthermore, because of time limitations, we did not follow-up APCs to determine the time-period until they become symptomatic after the initial diagnosis. Such information is essential to guide appropriate treatment strategies.

## Conclusion

The study shows that in the urban areas of Korhogo, in northern Côte d’Ivoire and Kaedi, in southern Mauritania, malaria prevalence is low with non-negligible proportions of APCs. We highlight also the implication of school-aged children and adults as *Plasmodium* spp. reservoirs and thus contributing greatly to the anchoring of malaria in favourable eco-epidemiological zones. Ours results have implications for malaria control programme in Côte d’Ivoire and Mauritania, which, currently, do not include APCs in intervention strategies likewise of school-aged children and adults. Hence, the authors recommend further investigations placing emphasis on APCs in each study country in order to design and implement appropriate surveillance and control measures.

## Additional file

Additional file 1: Multilingual abstracts in the five official working languages of the United Nations. (PDF 736 kb)

## Abbreviations

APC: Asymptomatic parasite carrier
*CI*: Confidence interval
GMPD: Geometric mean of parasite density
IPT: Intermittent preventive treatment
IRS: Indoor residual spraying
LLIN: Long-lasting insecticidal net
*OR*: Odd ratio
PD: Parasite density
RDT: Rapid diagnostic test
SD: Standard diagnostic
WHO: World Health Organization
