# Supplementary material for: Distribution of Plasmodium spp. infection in asymptomatic carriers in perennial and low seasonal malaria transmission settings in West Africa
Source: Infect Dis Poverty. 2018 Apr 25;7:39. doi: 10.1186/s40249-018-0412-9 (PMC5926534; doi:10.1186/s40249-018-0412-9)
Supplement: Supplementary file 1 — Multilingual abstracts in the five official working languages of the United Nations. (PDF 736 kb) [file 40249_2018_412_MOESM1_ESM.pdf]

Translation of the abstract into the six official working languages of the United Nations

توزّع عدوى المتصورة المنجلية لدى حامليها اللاعرضيين، هدف محتمل في أوساط انتقال الملاريا الحولي والموسمي المنخفض في غرب أفريقيا

كونستان ج. ن. جباليجبا، هامبات با، كيجبافوري د. سيلوي، عثمان با، إيمانويل تيا، محمدو شوايبو، ناثان ت. ي. تيان-بي، جريجوار ي. يابي، براما كونيه، يورغ أوتزنغر و بنجامين ج. كودو

الملخص

خلفية: منذ عام 2000، أحرز تقدم ملحوظ في الحدّ من الملاريا في جميع أنحاء العالم. إلا إن بعض البلدان في غرب أفريقيا ما تزال بؤرة لعدوى الملاريا مع إمكانية إصابة جميع الفئات العمرية.

يعتبر حاملو *Plasmodium spp* اللاعرضيون من المصادر المهمة للعدوى بالنسبة لنواقل الملاريا ، وهم بهذه الطريقة يسهمون في ترسيخ المرض في البيئات البيئية الوبائية المواتية.

تهدف هذه الدراسة إلى تقييم معدلات حالات الملاريا اللاعرضية في كورهوغو وكايدي ، وهما منطقتان حضريتان في شمال ساحل العاج وجنوب موريتانيا ، على التوالي.

الطرق: أجريت استقصاءات مقطعية خلال موسم الأمطار في عام 2014 وموسم الجفاف في عام 2015 في كلا المنطقتين. خلال كل موسم، اختيرت 728 أسرة عشوائياً وطُبّق استبيان أسري لجمع البيانات الديمغرافية والوبائية، يتضمن طرق الوقاية من الملاريا المستخدمة في المجتمعات. أخذت عينات الدم بوخز الاصبع لفحصها بيولوجيا باستخدام الفحص المجهري والاختبارات التشخيصية الروتينية السريعة (RDTs).

النتائج: أجري مسح شمل ما مجموعه 2672 أسرة و 15858 مشارك موافق. تم التأكد من حدوث العدوى بـ *Plasmodium* spp في 12.4 ٪ (ع = 832) و 0.3 ٪ (ع = 22) من الأفراد الذين شملهم التقييم في كورهوجو وكايدي، على التوالي.

في كورهوغو ، ارتبطت عدوى الملاريا اللاعرضية بشكل كبير بالعمر والموسم، مع وجود إمكانية أعلى بين عمري 5 - 14 سنة، وخلال موسم الأمطار.

في كايدي ، ارتبط خطر الإصابة بالملاريا اللاعرضية بالموسم فقط (أعلى خلال موسم الجفاف؛ cOR 6,37، 95%، الموثوقية: 1.87 - 21.63).

كانت المتصورة المنجلية *P. falciparum* هي النوع الغالب الذي حُدّد في كل من موقعي الدراسة ومثل 99.2 ٪ (ع = 825) من الأنواع التي حددت في كورهوغو و 59.1 ٪ (ع = 13) في كايدي.

لوحظت العِرسِيّات فقط في كورهوغو وخلال موسم الأمطار فقط بنسبة قدرها 1.3 ٪ (95 ٪ / الموثوقية: 0.7-2.4).

الاستنتاج: تظهر النتائج انتشارا منخفضا لنوبات الملاريا السريرية مع وجود نسبة كبيرة من حاملي العدوى اللاعرضيين في كلتا المنطقتين الحضريتين.

وتركز السياسات الوطنية لمكافحة عدوى الملاريا على علاج الحالات العرضية. يجب أن تكون استراتيجيات مكافحة الملاريا مصممة لرصد وتدبير عدوى الملاريا لدى حامليها اللاعرضيين.

يجب أن تكون استراتيجيات مكافحة الملاريا مصممة لرصد وتدبير عدوى الملاريا لدى حامليها اللاعرضيين.

وثمة حاجة ماسة لاتخاذ تدابير إضافية تشمل الرش الثمالي للأماكن المغلقة، والاستخدام الفعال للشبكات المشربة بالمبيدات الحشرية طويلة الأمد (LLINs) من أجل الحدّ من عدد حالات العدوى بـأنواع المنجليات في كورهوغو وكايدي.

Translated from English version into Arabic by Lina SM, proofread by Noor Alquraishi, through

TRANSLATORS WITHOUT BORDERS

西非常年和低季节性疟疾传播环境中无症状感染者中疟疾感染的分布

Constant G N Gbalégba, Hampâté Ba, Kigbafori D Silué, Ousmane Ba, Emmanuel Tia, Mouhamadou Chouaibou, Nathan T Y Tian-Bi, Grégoire Y Yapi, Brama Koné, Jürg Utzinger and Benjamin G Koudou

摘要

引言：2000年以来，全球在减少疟疾发病方面取得了重大进展。然而，西非一些国家仍然是疟疾感染的热点地区，所有年龄阶段的人群都面临感染风险。无症状疟原虫携带者是疟疾感染的重要来源，可通过传疟媒介在有利的生态流行病学环境中传播疟疾。本研究的目的是评估科特迪瓦北部和毛里塔尼亚南部的两个城市地区Korhogo和Kaedi的无症状疟疾病例感染情况。

方法：在2014年雨季和2015年旱季开展横断面调查。在每一季中，随机抽取728户家庭，发放家庭调查问卷，收集人口和流行病学数据，包括社区使用的疟疾预防方法。通过采集指尖血，使用显微镜镜检和常规的快速诊断试剂(RDTs)进行生物学检查。

结果：共计2672个家庭和15,858个参与者接受了调查。在Korhogo和Kaedi疟原虫感染率分别为12.4% (n = 832)和0.3% (n = 22)。在Korhogo，RDT检测的无症状疟疾发病率为10.5% (95% *CI*: 9.7–11.2)，而由显微镜检测的发病率为9.3% (95% *CI*: 8.6–10.0)。在Kaedi，显微镜检测的无症状疟疾发病率为0.2% (95% *CI*: 0.1–0.4)，而所有RDTs检测均为阴性(n = 8372)。在Korhogo，无症状疟疾感染与年龄和季节显著相关，5–14岁和雨季期间风险更高。在Kaedi，无症状疟疾感染的风险只与季节有关(在旱季较高；c*OR*：6.37, 95% *CI*: 1.87–21.63)。在Korhogo和Kaedi两个研究点发现的主要虫种为恶性疟原虫，分别为99.2% (n = 825)和59.1% (n = 13)。仅在Korhogo雨季观察到1.3% (95% *CI:* 0.7–2.4)的配子体。

结论：所研究两个城市地区的临床疟疾发病率低，有大量的无症状带虫者。国家针对疟疾感染的政策重点是治疗有症状的病例。疟疾防治策略应重视监测和管理无症状感染者，并需要采取其他措施，如室内残留喷洒，有效利用长效杀虫剂浸渍的蚊帐(LLINs)，以减少 Korhogo 和 Kaedi 疟原虫感染者的数量。

Translated from English version into Chinese by Xin-Yu Feng, edited by Pin Yang

CHINESE CENTER FOR DISEASE CONTROL AND PREVENTION
NATIONAL INSTITUTE OF PARASITIC DISEASES

Distribution des infections à *Plasmodium* spp. de porteurs sains pouvant constituer une cible dans le cadre de la transmission perannuelle et en basse saison du paludisme en Afrique de l'Ouest

Constant G N Gbalégba, Hampâté Ba, Kigbafori D Silué, Ousmane Ba, Emmanuel Tia, Mouhamadou Chouaibou, Nathan T Y Tian-Bi, Grégoire Y Yapi, Brama Koné, Jürg Utzinger et Benjamin G Koudou

Résumé

Contexte: Bien que des progrès considérables aient été réalisés dans le monde entier depuis 2000 dans la lutte contre le paludisme, certains pays d'Afrique de l'Ouest restent des zones à haut risque d'infection pour tous les groupes d'âge. Les porteurs sains de *Plasmodium* spp. constituent des sources importantes d'infection pour les vecteurs du paludisme et contribuent à la pérennité de la maladie dans

les environnements éco-épidémiologiques qui lui sont favorables. L'objectif de la présente étude était d'évaluer le taux de cas de paludisme asymptomatiques à Korhogo et Kaédi, deux zones urbaines situées l'une dans le nord de la Côte d'Ivoire et l'autre dans le sud de la Mauritanie.

Méthodes: Des études transversales ont été réalisées dans les deux régions au cours de la saison des pluies 2014 et de la saison sèche 2015. Au cours de chaque saison, 728 foyers ont été choisis au hasard et un questionnaire a été administré à chacun afin de recueillir des données démographiques et épidémiologiques, notamment sur les méthodes de prévention du paludisme employées par les populations. Des échantillons de sang ont été prélevés par ponction du doigt en vue d'un examen biologique par microscopie et tests de diagnostic rapide (TDR) de routine.

Résultats: Au total, 2672 foyers et 15 858 participants consentants ont été inclus dans l'enquête. L'infection par *Plasmodium* spp. a été confirmée chez 12,4 % ($n = 832$) des habitants de Korhogo et 0,3 % ($n = 22$) de ceux de Kaédi. La prévalence du paludisme asymptomatique à Korhogo était de 10,5 % (IC à 95 % : 9,7–11,2) avec les examens au microscope et 9,3 % (IC à 95 % : 8,6–10,0) avec les TDR. À Kaédi, la prévalence du paludisme asymptomatique était de 0,2 % (IC à 95 % : 0,1-0,4) avec la microscopie et que tous les TDR effectués se sont avérés négatifs ($n = 8372$). À Korhogo, l'infection palustre asymptomatique était significativement associée à l'âge et à la saison, avec un risque plus élevé chez les 5-14 ans et pendant la saison des pluies. À Kaédi, le risque d'infection asymptomatique était associé à la saison uniquement (plus élevé pendant la saison sèche ; RC bruts (cOR) 6,37, IC à 95 % : 1,87–21,63). *P. falciparum* est l'espèce prédominante identifiés dans les deux sites d'étude, où elle représentait 99,2 % des infections ($n = 825$) à Korhogo et 59,1 % ($n = 13$) à Kaédi. Nous n'avons observé des gamétocytes qu'à Korhogo, et uniquement pendant la saison des pluies, dans 1,3 % des cas (IC à 95 % : 0,7–2,4).

Conclusion: Nos résultats montrent une faible prévalence des épisodes de paludisme clinique et une proportion significative de porteurs sains dans les deux zones urbaines étudiées. Or les politiques nationales de lutte contre les infections palustres se concentrent sur le traitement des cas symptomatiques. Des stratégies de lutte doivent être définies pour surveiller et traiter les infections palustres des porteurs sains. Des mesures supplémentaires, notamment la pulvérisation résiduelle intérieure et l'utilisation efficace des moustiquaires imprégnées d'insecticide de longue durée (MILD), sont très nécessaires pour réduire le nombre d'infections à *Plasmodium* spp. à Korhogo et Kaédi.

Translated from English version into French by Suzanne Assenat, proofread by Oceane Hernandez, through

TRANSLATORS
WITHOUT BORDERS

Распространение инфекции, вызванной *малярийными видами плазмодиев,* у бессиптомных носителей, которые являются потенциальной мишенью в многолетней перспективе и в условиях низкой сезонной передачи малярии в Западной Африке

Констант Г. Н. Бгалегба, Ампате Ба, Кигбафори Д. Силуэ, Усман Ба, Эмманюэль Тиа, Мухамаду Шуэбу, Натан Т. И. Тиан-Би, Грегуар И. Япи, Брама Конэ, Юрг Утцингер и Бенджамин Г. Куду

Реферат

Справочная информация: С 2000 года во всем мире был достигнут значительный прогресс в снижении малярии. Однако некоторые страны в Западной Африке по-прежнему являются очагами заражения малярией, при этом в группу риска попадают все возрастные категории. Бессимптомные носители *малярийных видов* плазмодиев являются важными источниками инфицирования переносчиков малярии и, таким образом, способствуют закреплению заболевания в благоприятной экоэпидемиологической среде. Цель настоящего исследования заключалась в оценке бессимптомных случаев случаев заболеваемости малярией в Корхого и Каэди, двух городских районах, расположенных, соответственно, на севере Кот-д'Ивуара и на юге Мавритании.

Методы: В сезон дождей 2014 года, а также в сухой период 2015 года в обоих местностях были проведены перекрёстные обследования. В течение каждого из указанных периодов случайным образом были отобраны 728 домохозяйств, по которым была распространена анкета для домохозяйств с целью сбора демографических и эпидемиологических сведений, в том числе о профилактических методах борьбы с малярией на уровне сообществ. Для проведения биологического обследования с помощью микроскопии и типовых диагностических экспресс-тестов (ДЭТ) были взяты анализы крови из пальца.

Результаты: В целом было обследовано 2 672 домохозяйств и 15 858 участников, давших своё согласие. *Инфекция, вызванная плазмодием малярийного вида* была выявлена у 12,4% (*n* = 832) и 0,3% (*n* = 22) обследованных лиц в Корхого и Каэди соответственно. В Корхого распространённость бессимптомной малярии составила 10,5% (95% *CI:* 9,7-11,2), согласно результатам обследования с помощью микроскопии и 9,3% (95% *CI:* 8.6-10.0) при методе ДЭТ. В Каэди распространённость бессимптомной малярии находилась на уровне 0,2% (95% *CI*: 0.1–0,4) согласно микроскопии, тогда как все исследования, выполненные методом ДЭТ дали отрицательные результаты (*n* = 8372). В Корхого инфицированность бессимптомной малярией в значительной степени носила сезонный и возрастной характер, где в группу более высокого риска входили дети от 5 до 14 лет в сезон дождей. В Каэди риск бессимптомной инфицированности малярией был связан исключительно с сезонной составляющей (повышаясь в сухой период; предварительно *OR* (с*OR*), 6,37; 95% *CI*: 1,87–21,63). *P. falciparum* был преобладающим видом, обнаруженным в обоих регионах исследования, и составил 99,2% (*N* = 825) в Корхого и 59,1% (*N* = 13) в Каэди. Гаметоциты наблюдались только в Корхого и только в сезон дождей на уровне 1,3% (95% *CI*: 0,7-2,4).

Заключение: Результаты демонстрируют низкую распространённость клинических случаев малярии, значительная доля которых приходится на бессимптомных носителей заболевания в обоих городских районах. Государственная политика по борьбе с заболеваемостью малярией ориентирована на лечение симптоматических случаев. Необходимо разработать стратегии по борьбе с малярией с целью мониторинга и контроля инфекции у бессимптомных носителей. Для снижения инфекций, *вызванных малярийными видами* плазмодиев, в Корхого и Каэди категорически необходимо внедрение дополнительных мер, таких как опрыскивание

внутренних жилых помещений инсектицидами и эффективное применение надкроватных сеткок, обработанных инсектицидом длительного действия (СОИДД).

Translated from English version into Russian by Liudmila Tomanek, proofread by Ekaterina_Rugg, through

Distribución de la infección de *Plasmodium* spp. en portadores asintomáticos, un objetivo potencial en entornos de transmisión del paludismo perenne y de baja estacionalidad en África Occidental

Constant G N Gbalégba, Hampâté Ba, Kigbafori D Silué, Ousmane Ba, Emmanuel Tia, Mouhamadou Chouaibou, Nathan T Y Tian-Bi, Grégoire Y Yapi, Brama Koné, Jürg Utzinger y Benjamin G Koudou

Resumen

Antecedentes: desde el año 2000, se han logrado progresos sustanciales en la reducción del paludismo en todo el mundo. Sin embargo, algunos países de África Occidental siguen siendo un foco de infecciones por paludismo con todos los grupos de edad en riesgo. Los portadores asintomáticos de *Plasmodium* spp. son fuentes importantes de infección para los vectores del paludismo y, por lo tanto, contribuyen a anclar la enfermedad en entornos eco-epidemiológicos favorables. El objetivo de este estudio fue evaluar las tasas de casos asintomáticos de paludismo en Korhogo y Kaedi, dos zonas urbanas del norte de Costa de Marfil y el sur de Mauritania, respectivamente.

Métodos: Se llevaron a cabo encuestas transversales durante la temporada de lluvias en 2014 y la temporada seca en 2015 en ambos entornos. Durante cada temporada, se seleccionaron al azar 728 hogares y se aplicó un cuestionario basado en los hogares para recopilar datos demográficos y epidemiológicos, incluidos los métodos de prevención del paludismo utilizados en las comunidades. Se obtuvieron muestras de sangre con pinchazo en los dedos para su examen biológico mediante microscopia y pruebas de diagnóstico rápido de rutina (DRR).

Resultados: En total, se encuestaron 2672 hogares y 15 858 participantes con consentimiento. La infección por*Plasmodium* spp. se confirmó en 12,4 % (*n* = 832) y en 0,3 % (*n* = 22) de los individuos analizados en Korhogo y Kaedi, respectivamente. En Korhogo, la prevalencia del paludismo asintomático fue del 10,5 % (95 % *CI:* 9,7–11,2) según se determinó por microscopia y de 9,3 % (95 % *CI:* 8,6–10,0) cuando se evaluó por DRR. En Kaedi, la prevalencia del paludismo asintomático fue de 0,2 % (95 % *CI*: 0,1–0,4) de acuerdo con la microscopia, mientras que todos los DRR llevados a cabo fueron negativos (*n* = 8372). En Korhogo, la infección asintomática por paludismo se asoció significativamente con la edad y la estación del año, con un mayor riesgo entre los 5 y los 14 años de edad, y durante la estación de lluvias. En Kaedi, el riesgo de infección por paludismo asintomático se asoció solo con la temporada (mayor durante la temporada seca; aproximadamente *OR* (c*OR*), 6,37, 95 % *CI*: 1,87–21,63). *P. falciparum* fue la especie predominante identificada en ambos sitios de estudio que

representan el 99,2 % (*n* = 825) en Korhogo y 59,1 % (*n* = 13) en Kaedi. Los gametocitos se observaron solo en Korhogo y solo durante la temporada de lluvias en un 1,3 % (95 % *CI*: 0,7–2,4).
Conclusión: los resultados demuestran una baja prevalencia de episodios de paludismo clínico con una proporción considerable de portadores asintomáticos en ambas zonas urbanas. Las políticas nacionales para las infecciones por paludismo se centran en el tratamiento de casos sintomáticos. Las estrategias de control del paludismo deben diseñarse para monitorear y manejar las infecciones de paludismo en portadores asintomáticos. Se necesitan medidas adicionales, incluida la fumigación residual en interiores, el uso efectivo de redes impregnadas con insecticidas de larga duración (LLIN), que son muy necesarias para reducir el número de infecciones por *Plasmodium* spp. en Korhogo y Kaedi.

Translated from English version into Spanish by Ilduara Escobedo, proofread by Ana Usán, through

TRANSLATORS
WITHOUT BORDERS
